# Declining trend in HIV new infections in Guangxi, China: insights from linking reported HIV/AIDS cases with CD4-at-diagnosis data

**DOI:** 10.1186/s12889-020-09021-9

**Published:** 2020-06-12

**Authors:** Xiaodan Sun, Wenmin Yang, Sanyi Tang, Mingwang Shen, Tianyang Wang, Qiuying Zhu, Zhiyong Shen, Shuai Tang, Huanhuan Chen, Yuhua Ruan, Yanni Xiao

**Affiliations:** 1 School of Mathematics and Statistics, Xi’an Jiaotong University, Xi’an, 710049 China; 2 Guangxi Center for Disease Control and Prevention, Nanning, China; 3 School of Mathematics and Information Science, Shaanxi Normal University, Xi’an, China; 4 Department of Epidemiology and Biostatistics, School of Public Health, Xi’an Jiaotong University Health Science Center, Xi’an, China; 5 State Key Laboratory of Infectious Disease Prevention and Control (SKLID), Collaborative Innovation Center for Diagnosis and Treatment of Infectious Diseases, Chinese Center for Disease Control and Prevention (China CDC), Beijing, China

**Keywords:** HIV/AIDS, New infections, Undiagnosed infections, Diagnosis rate, CD4-stage structured model

## Abstract

**Background:**

The Guangxi Zhuang Autonomous Region bears a relatively high burden of HIV/AIDS infection. The number of accumulatively reported HIV/AIDS cases in Guangxi is the third highest among 31 provinces or Autonomous Region from 2004 to 2007, changed to the second highest between 2011 and 2013, then returned to the third highest again after 2014. We aim to estimate the new infections and evaluate the real-time HIV epidemic in Guangxi, China, in order to reveal the rule of HIV transmission.

**Methods:**

Firstly, the number of annually reported HIV and AIDS cases, as well as the number of cases linked with CD4 data are extracted from the HIV/AIDS information system in China. Secondly, two CD4-staged models are formulated by linking the with-host information on CD4 level to between-host transmission and surveillance data. Thirdly, new HIV infections, diagnosis rates and undiagnosed infections over time are estimated by using Bayesian method and Maximum Likelihood Estimation method.

**Results:**

The data reveal that the newly reported cases have been decreasing since 2011, while lots of cases are identified at late CD4 stage. The data fitted results indicate that both models can describe the trend of the epidemic well. The estimation results show that the new and undiagnosed infections began to decrease from the period2006 - 2008. However, the diagnosis probabilities/rates keep at a very low level, and there are still a large number of infections undiagnosed, most of which have a large probability to be identified at late CD4 stage.

**Conclusions:**

Our findings suggest that HIV/AIDS epidemic in Guangxi has been controlled to a certain extent, while the diagnosis rate still needs to be improved. More attentions should be paid to identify infections at their early CD4 stages. Meanwhile, comprehensive intervention measures should be continually strengthened in avoid of the rebound of new infections.

## Background

The Guangxi Zhuang Autonomous Region bears a relatively high burden of HIV/AIDS infection. Since the first local case was detected in 1996, HIV has been spreading in Guangxi. The number of accumulatively reported HIV/AIDS cases in Guangxi is the third highest among 31 provinces or Autonomous Region from 2004 to the end of 2007 [1, 2], changed to the second highest between 2011 and 2013 [3–5], then returned to the third highest again after 2014 [6]. Due to particular geographic location and population characteristics with diverse risk groups [7, 8], HIV infections in Guangxi exhibit its traits including the large number of accumulatively reported cases [1–6], high prevalence among general population [9], diversified transmission routes and risk groups and etc [10–12]. Since the Four Free and One Care policy initiated in 2003, Chinese government has advocated lots of prevention and control strategies [1, 2, 13–16], which greatly enhanced HIV prevention and control. It is observed that the number of annually reported cases shows the decline trend in recent years, however the current HIV incidence (or new HIV infections) in Guangxi still remains unclear mainly because of the long latent period of the HIV infected cases [17]. Hence, getting a high-quality evaluation for the HIV epidemic in Guangxi, and possibly identifying the key process or parameters that significantly affect HIV infection fall within the scope of this study.

Lots of experts has been devoting to the estimation of HIV new infections and HIV undiagnoses in recent years [18–20], but accurate estimation of real-time HIV infection in China becomes challenging because of the differences in various transmission routes, patterns of risk behaviours, the intensities of interventions for various regions, high loss ratio of follow-up and etc [1, 21]. HIV epidemic in Guangxi, representing national wide pattern of HIV infection to some extent, has new features including quick increase of HIV infections caused by heterosexual transmission [2, 9–12, 22], increasing of reported cases with age older than 50 [10] and regional itself characteristics, which will bring new challenges to the estimation. A great number of dynamic and statistical models has been formulated to estimate HIV new infection and investigate HIV epidemic trends [23–28]. These estimations have been mainly based on the surveillance data on population level without including official clinic data at individual level. However, both transmission probability and disease-related mortality are dependent on within-host viral loads and CD4 level and hence greatly affect the estimation of new infections [29]. How to nest the with-host information on viral loads and CD4 level into between-host modelling and simulations and accurately estimating HIV new infections becomes challenging.

The main purpose of this study is to focus on the Guangxi HIV infection and estimate the new infections and evaluate the real-time HIV epidemic in Guangxi Zhuang Autonomous Region, China. According to the characteristics of China’s HIV/AIDS data we extend the models, proposed by Birrell et al. [30] and van Sighem et al. [31], and formulate two different models based on the same CD4-staged structure. We fully use data on the population level as well as the data on the individual level to estimate the new infections, the diagnosis probabilities/rates at different stages as well as the undiagnosed infections.

## Methods

**Fig. 1:**
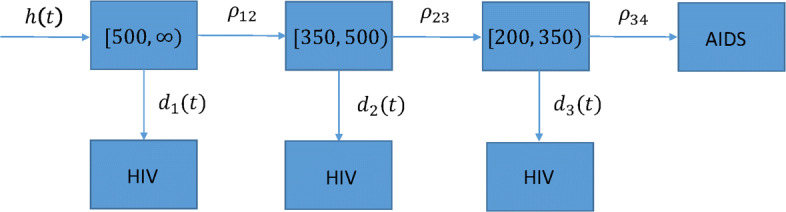
The CD4-staged modelling structure. New infections happen at rate *h*(*t*), then progress through each HIV stage until AIDS stage with progression rate denoted by *ρ*_*ij*_(*t*). HIV cases may be diagnosed at any HIV stage, with diagnosis probability of *d*_*i*_(*t*) or be diagnosed until AIDS stage

In this article two different methods, i.e. Markov model and deterministic compartmental model which based on the same CD4-staged structure, as shown in Fig. 1, are used to estimate the time series new infections and undiagnoses in Guangxi. For the first method, we estimate the unknown variables involved in the model using the CD4-based Bayesian back-calculation, and conduct the posterior inference to obtain the posterior distributions of new infections, diagnosis probabilities as well as the undiagnosed infections over time. For the second method, we solve the deterministic model numerically and estimate the unknown variables using the maximum likelihood estimation method, then calculate the number of new HIV infections, undiagnosed infections as well as the diagnosis rates with 95% confidence intervals obtained by the parametric bootstrap method.

The model framework, as shown in Fig. 1, is extended based on those proposed by Birrell et al. [30] and van Sighem et al. [31]. Due to the fact that both the HIV infected cases with CD4 cell counts less than 200 and those with symptoms are classified as AIDS patients in China, HIV infected individuals progress three CD4 stages, i.e. [500,*∞*),[350,500), [200,350), then to AIDS stage. It is assumed that new infections happen at rate *h*(*t*) at time *t*, and HIV cases can be diagnosed at any CD4 stage, the diagnosis rate at CD4-stage *i* at time *t* is denoted by *d*_*i*_(*t*). It is further assumed that the progression rates between different stages are known and each individual has the same probability to get CD4 measurements within 3 months after diagnosis. In this article, three different groups of disease progression rate *ρ*_*i*,*i*+1_ are adopted. The progression rates are firstly chosen according to Zhou et al. [32] who supposed that the disease stays in each stage for 3 years, denoted by progression rate 1 (PR1). Then, progression rates are chosen as those used in Birrell et al. [30] and van Sighem et al. [31], in which the waiting times in three stages are 2.56, 2.17, 2.16 (denoted by progression rate 2 (PR2)) and 6.37, 2.86, 3.54 (denoted by progression rate 3 (PR3)), respectively. In both methods, PR1 is initially chosen to show the main estimation results, then sensitivity analysis is implemented by comparing outcomes for various progression rates.

As the first case was identified in 1996 in Guangxi, it is supposed that the first HIV case may be infected as early as 1990 considering the long latent period of HIV infection. Thus, the annually number of new infections and undiagnosed infections from 1990 to 2017, and diagnosis probabilities from 1996 to 2017 (as there is no infections diagnosed before 1996) will be estimated. In the following, we shall introduce how we estimate these quantities by applying the methods adopted by Birrell et al. [30] and van Sighem et al. [31], respectively.

### Method 1

Markov model is formulated with the model framework shown in Fig. 1. According to Birrell’s method [30], the likelihood function is contributed by two parts: HIV/AIDS diagnosis data and CD4-at-diagnosis data, see the details in Additional file 1. The hierarchical Bayesian approach is used which includes a random walk specification for the incidence and diagnosis curves. Thus, both the number of new infections and diagnosis probabilities have autocorrelation which could improve the identifiability. Particularly, logit-diagnosis probabilities and log-infection rates are adopted (see details in Additional file 1). Since all HIV and AIDS cases are reported as long as diagnosed according to our reporting policy, the effect of under-reporting is not considered.

As national sentinel surveillance began to implement since 1995 [13], a factor *c*_90_ is introduced to represent the reduced diagnosis before 1995(see details in Additional file 1). The diagnosis probabilities after 1995 are further divided into 3 stages, 1995 - 2003, 2004 - 2010 and 2011 - 2017, due to the following reasons. Firstly, the Four Free and One Care policy addressed at the end of 2003 from when China began to offer free HIV testing, which may increase the case finding rate and affect the precision of the random walk [33]. Secondly, Guangxi AIDS Conquering Project (GACP) initiated in 2011 [16] may further increase the diagnosis probability. These two policies could also affect the HIV infection rate greatly. Furthermore, the needle exchange programme was initiated in Guangxi from 1999 [13], and the outbreak of severe acute respiratory syndrome (SARS) in 2003 inspired the government to take more attention on public-health issues [13]. Therefore, for estimating the infection rate we further split the epidemic into four stages, i.e. 1990 - 1998, 1999 - 2003, 2004 - 2010 and 2011 - 2017. The precision of the random-walk for the logit-diagnosis probabilities and log-infection rates are then piecewise constants according to the corresponding different stages. Also, we follow the prior distribution for the random-walk variances used in Birrell et al. [30] which are based on the results of De Angelis et al. [34].

Based on the prior distribution and likelihood function mentioned above, we estimated the posterior distributions by the MCMC method, which is conducted using the rjags package for R.

### Method 2

A deterministic model is proposed based on the CD4-staged structure shown in Fig. 1, see Additional file 1 for details. The basic idea of the method is similar to those used by van Sighem et al. [31] with the estimation procedure being slightly different. Since new infections and diagnosis rate may not always change much within successive years, both new infections and diagnosis rates are supposed to be step functions, which can also reduce the number of parameters to be estimated. More precisely, new infections for successive 3 years and diagnosis rates for successive 4 or 5 years except for 2005 and 2011 are constants. The diagnosis rates in 2005 and 2011 are special and much larger compared with those of other years due to the fact that nationwide testing of high-risk groups was implemented in 2004 and 2005 [35] and the GACP was initiated in 2011 [16]. Thus, diagnosis rates in 2005 and 2011 are assumed to be constants. Meanwhile, the diagnosis rate in the first 6 years is assumed to be zero, since there is no diagnosis from 1990 to 1995. The step functions of new infections and diagnosis rates are given in Additional file 1.

It is supposed that the number of new diagnosed HIV, AIDS cases and HIV cases linked with CD4 counts at each CD4 stage follow Poisson distributions. Then the mean value of incidence *h*(*t*), diagnosis rate *d*_*i*_(*t*) as well as the undiagnosed HIV-positives can be estimated by using the Maximum Likelihood Estimation method. The 95% confidence intervals (CI) of the estimations are obtained by using the parametric bootstrap method, in which the new datasets for each year and for each data items are resampled from Poisson distributions with the mean value defined by the model. 200 new data sets are resampled, and the model is refitted to each of the new datasets. Based on these 200 and estimation results, 95% CI can be determined as the 2.5th and 97.5th percentiles.

### The data

We extract the needed data from three databases: i) case report database which includes the ID card number, disease stages (i.e. HIV-positive or AIDS stage) when diagnosed, ‘date of recordance’ (i.e. the date for the HIV or AIDS cases being diagnosed), ‘date of HIV to AIDS’ (i.e. the date for HIV-positve individual progresses to AIDS stage) etc. ii) follow-up database which includes the ID card number, follow-up times, date of follow-up, date of CD4 testing, CD4 testing result, etc. iii) treatment database which includes the ID card number, treatment number, treatment follow-up times, date of treatment follow-up, treatment regimen, etc. All these three databases are obtained from HIV/AIDS information system in China. It is worth noting that these three databases can be connected to each other according to the ID card number and treatment number.

According to the model and likelihood function, we need to get the number of new observed HIV (**D**={*D*_*j*_:*j*=1,⋯,*N*}) and AIDS cases (**A**={*A*_*j*_:*j*=1,⋯,*N*}) in each time interval, the observed number of infected cases with CD4-at-diagnosis falling into each CD4 group ***C***_*j*_=(*C*_1,*j*_,*C*_2,*j*_,*C*_3,*j*_) and the number of observed cases linked with CD4 counts at diagnosis $\textit {\textbf {N}}_{j}=\sum _{k}^{3} C_{k,j}$ in each time interval. These necessary data are extracted according to the following approximations (denoted by *APPR*_*i*_) and procedure (denoted by *PROC*_*i*_):

- (*APPR*_1_:) for each case, if the ‘date of HIV to AIDS’ falls within 3 months of the ‘date of recordance’, then the case is classified as AIDS patient when diagnosed.
- (*APPR*_2_:) for each case, if the first ‘date of CD4 testing’ (i.e. when the corresponding ‘follow-up times’ in follow-up database is 1st) is before the first ‘date of treatment follow-up’ (i.e. when the corresponding ‘treatment follow-up times’ in treatment follow-up database is 1st), it should be thought that the first ‘CD4 testing result’ was done before the initiation of treatment.
- (*APPR*_3_:) for each case, if the ‘date of CD4 testing ’ falls within 3 months of the ‘date of recordance’, it should be thought that the corresponding ‘CD4 testing result’ is the CD4-at-diagnosis.
- (*PROC*_1_:) According to the ‘date of recordance’, ‘disease stages’ and the above classification, we calculate the number of new observed HIV and AIDS cases in each time interval.
- (*PROC*_2_:) According to the CD4-at-diagnosis for certain HIV cases, we divide HIV cases into three CD4 groups, combining the ‘date of recordance’ for each case, we then get the number of HIV cases at each CD4 stage in each time interval. And the number of observed cases linked with CD4 counts at diagnosis is further obtained.

**Fig. 2:**
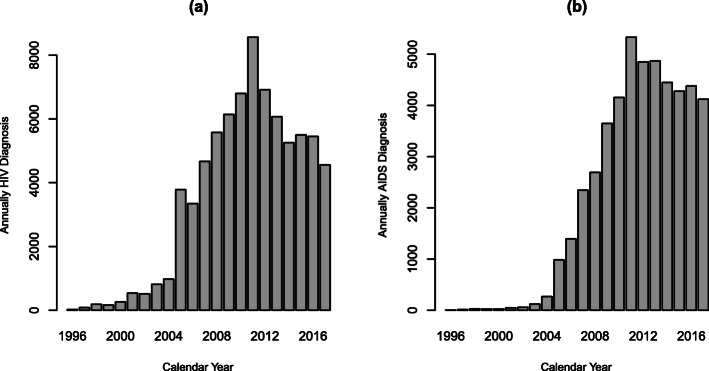
The annually number of new HIV and AIDS diagnosis from year 1996 to 2017. **a** the annually number of new HIV diagnosis; **b** the annually number of new AIDS diagnosis

The data indicates that the accumulated reported number of HIV infected cases and AIDS patients is 124,282 by the end of 2017. On the basis of the approximations and procedure we obtain the number of annually HIV and AIDS diagnosis from year 1996 to 2017 as shown in Fig. 2, which indicates that both the numbers of annually HIV and AIDS diagnosis increase gradually from 1996, then quickly increase around 2005 and peak in 2011, and finally decrease slowly.

**Fig. 3:**
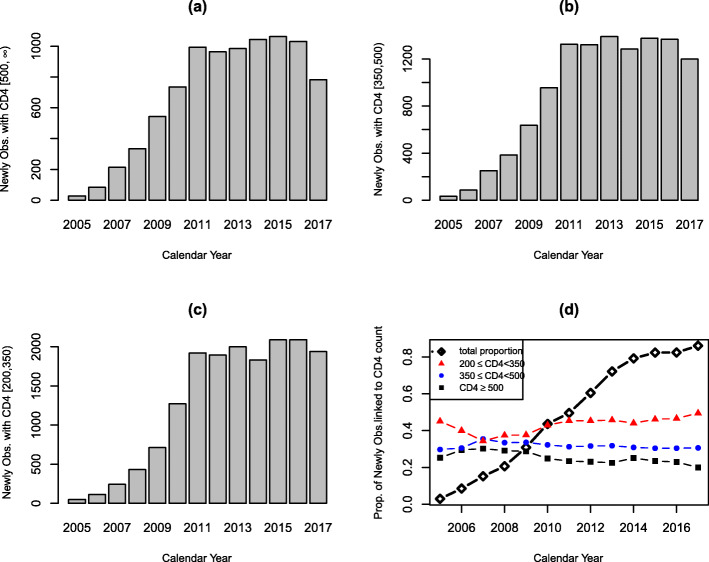
The annually number of new HIV diagnosis linked with CD4 cell counts. **a** newly observed cases at CD4 stage [500, *∞*); **b** newly observed cases at CD4 stage [350,500); **c** newly observed cases at CD4 stage [200,350); **d** black hollow squares are the proportion of HIV cases linked with CD4 counts among all the observed HIV cases over time, red triangles, blue circles and black solid squares are the proportion of HIV cases at each CD4 stage among those cases linked with CD4 counts

Note that there is a big jump on both the HIV and AIDS diagnosis data in 2005 and 2010, which is because the nationwide testing program for high-risk groups was implemented in 2004 and 2005 [35] and the State Council of China issued ‘Notice of the State Council on further strengthening AIDS prevention and control work’ at the end of 2010 [14]. Only after 2005 cases began to receive CD4 test once diagnosed with HIV, thus the CD4-at-diagnosis data have been available since 2005. The number of annual HIV cases linked with CD4 counts in each stage continually increases, as shown in Fig. 3. More precisely, the proportion of HIV cases who are linked with CD4 counts increases from 3% in 2005 to 86% in 2017, as shown Fig. 3d. It is interesting to observe that almost half HIV cases linked with CD4 counts were identified at the third CD4 stage, i.e. 200≤*CD*4<350, as illustrated by red triangles in Fig. 3d.

## Results

When progression rate 1 is chosen, the data fitted results by method 1 and 2 are illustrated in Additional files 2 and 3, respectively, where solid and dotted curves denote the expected value and 95% CI (Credible or Confidence Interval) estimated by the models, dark squares, circles and triangles denote the observed data. The fitted results show that both models can describe the trend of the epidemic well, including the number of annual HIV/AIDS diagnosis and annual HIV cases linked with CD4 counts in each stage. Especially, almost all the observed data fall within the estimated 95% CI obtained by the first method, which indicates that our estimation can well reflect the actual situation. In the following, we initially give the results obtained by the first method.

**Fig. 4:**
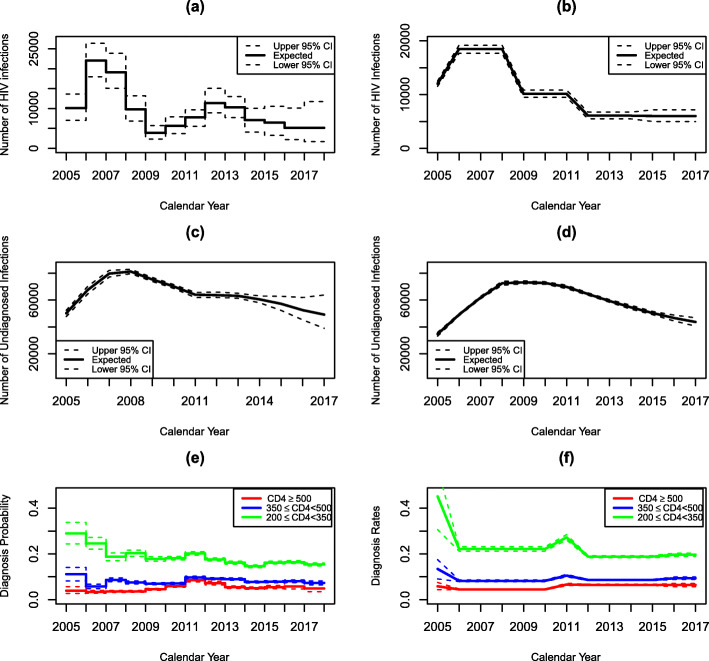
Yearly new infections **a**-**b**, Undiagnosed prevalence **c**-**d**, Diagnosis probabilities **e**-**f** from 2005 to 2017. The left panel, i.e. **a**, **c** and **e**, and the right panel, i.e. **b**, **d** and **f**, illustrate the results estimated by method 1 and 2, respectively. The waiting time in each CD4 stage are 3, 3, 3 years, respectively. Diagnosis probabilities in CD4 stage [500, *∞*), [350,500) and [200,350) are denoted by red, blue and green, respectively. The dotted lines give the 95% CI

Figure 4a and Table 1 give the number of new HIV infections, which varies with time. Overall, the number of new infections decreases from year 2006, accompanied by a short time increase between 2009 and 2012, then continually decreases until 2017. In fact, the new infection reaches the peak value in 2006 with 22066 (95% Credible Interval (CI) [17976, 26353]) cases, and drops to the lowest value in 2009 with 3876 (95% CI [2337, 5709]) cases. The new infection rebounds between 2009 and 2012, and comes up to 11384 (95% CI [8923, 15069]) cases in 2012, then drops to 5134 (95% CI [1677, 11718]) cases in 2017. It is worth mentioning that the uncertainty for the estimation becomes larger and larger due to less and less information being available for recent infections [18]. The number of new infections from year 1990 to 2017 is shown in Additional file 4.

**Table 1:** Yearly new infections for different progression rates

Year	Progression rate 1		Progression rate 2		Progression rate 3	
	method 1	method 2	method 1	method 2	method 1	method 2
2005	10100	12023	8290	8337	14562	21515
2006	22066	18480	16665	15650	38765	24192
2007	19115	18480	15176	15650	10468	24192
2008	9816	18480	18102	15650	3068	24192
2009	3876	10126	4466	12343	1751	3465
2010	5676	10126	8614	12343	2495	3465
2011	7786	10126	8427	12343	4197	3465
2012	11384	6083	10633	7134	8486	4177
2013	10284	6083	10037	7134	10924	4177
2014	7075	6083	7094	7134	7526	4177
2015	6450	6013	6423	7445	6130	3205
2016	5155	6013	5532	7445	5221	3205
2017	5134	6013	5654	7445	5179	3205

The curve of the undiagnosed infections, shown in Fig. 4c, has a sharp raise from 2005 and reaches the peak value of 81224 (95% CI [79652, 82878]) cases in 2008, as Table 2 shows. Fortunately, the undiagnosed infections begin to decrease from 2008 with relatively low declining slop around 2012, gradually continue to decline to 49203 (95% CI [39015, 63783]) cases in 2017. Similar with what we observed in the estimation of new infections, the uncertainty becomes large for recent years. Although the undiagnosed infections show decline trend in recent years, there are still lots of HIV infections undiagnosed. More than 40 thousands infections are still undiagnosed by 2017. The number of undiagnosed infections from year 1990 to 2017 can be found in Additional file 4.

**Table 2:** Undiagnosed infections for different progression rates

Year	Progression rate 1		Progression rate 2		Progression rate 3	
	method 1	method 2	method 1	method 2	method 1	method 2
2005	50162	34717	36310	22089	84900	68141
2006	67548	49199	48262	33941	118935	88210
2007	79676	61809	56428	43846	122487	106442
2008	81224	72496	66261	51739	117359	122772
2009	75370	73167	60939	54554	109367	116715
2010	70127	72701	58596	56108	100983	109573
2011	63992	69791	53081	55564	91342	99831
2012	63677	64474	51971	51369	88304	92675
2013	63116	59295	51105	47201	88408	85667
2014	60501	54562	48510	43513	86426	79072
2015	57217	50378	45155	40803	82792	72087
2016	52507	46680	40820	38718	78241	65506
2017	49203	43704	37845	37247	74889	59703

Though the new infections as well as the undiagnosed cases decrease after 2008, the diagnosis probabilities do not change much in recent 13 years, as shown in Fig. 4e and Table 3. More precisely, the diagnosis probability at the late CD4 stage, i.e. less than 350, decreases from 0.246 (95% CI[0.220,0.272]) in 2006 to 0.154 (95% CI[0.147,0.161]) in 2017. While, the diagnosis probability at CD4 stage [350,500) varies between 0.06 to 0.11 during the recent 13 years. It is worth pointing out that the probability for HIV infections being diagnosed at early CD4 stage, i.e. more than 500, generally increases from 0.034 (95% CI[0.028,0.040]) in 2006 to 0.050 (95% CI[0.035,0.070]) in 2017. Meanwhile, it is obvious that diagnosis probabilities in all the three CD4 stages jump to higher values in 2005 and 2011, which is in agreement with the data shown in Fig. 3. But, the overall diagnosis probabilities for HIV infections are still very low, especially at early CD4 stages. The diagnosis probabilities from year 1996 to 2017 can be found in Additional file 4.

**Table 3:** Diagnosis probabilities at different CD4 stages for progression rate 1

Year	CD4 Stage [500,*∞*)		CD4 Stage [350,500)		CD4 Stage [200,350)	
	method 1	method 2	method 1	method 2	method 1	method 2
2005	0.039	0.059	0.112	0.134	0.290	0.451
2006	0.034	0.045	0.058	0.082	0.246	0.222
2007	0.036	0.045	0.087	0.082	0.187	0.222
2008	0.037	0.045	0.075	0.082	0.203	0.222
2009	0.046	0.045	0.070	0.082	0.178	0.222
2010	0.059	0.045	0.072	0.082	0.181	0.222
2011	0.085	0.066	0.097	0.105	0.202	0.272
2012	0.072	0.065	0.091	0.086	0.176	0.188
2013	0.054	0.065	0.090	0.086	0.161	0.188
2014	0.050	0.065	0.078	0.086	0.146	0.188
2015	0.054	0.065	0.079	0.086	0.162	0.188
2016	0.058	0.064	0.080	0.093	0.164	0.196
2017	0.050	0.064	0.073	0.093	0.154	0.196

**Fig. 5:**
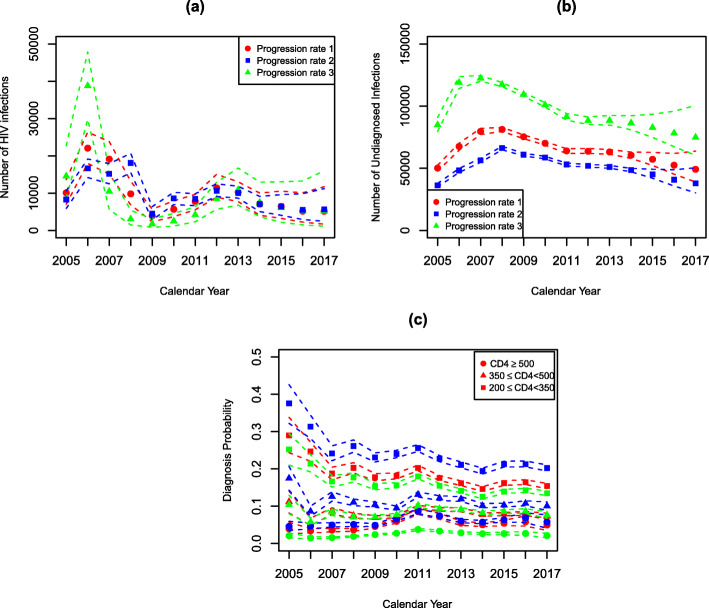
The comparison of new infections **a**, undiagnosed infections **b**, and diagnosis probabilities **c** by three different groups of progression rates (estimated by method 1). The waiting time in each CD4 stage for red, blue and green are PR1:3, 3, 3; PR2: 2.56, 2.17, 2.16 and PR3: 6.37, 2.86 3.54, respectively. The dotted lines give the 95% CI

We change the progression rates and implement the estimation again to examine the influence of the progression rate on our main results. The comparison between the new infections for three different groups of progression rate are illustrated in Fig. 5a and Table 1. Clearly, all these three estimations reveal the similar variation trends of the new infections. Furthermore, the estimations for new infections from year 2013 to 2017 are relatively close to each other. It is interesting to observe that low progression rate, corresponding to long waiting time, induces great new infections and undiagnosed infections with great variations. This indicates slow progression in HIV dynamics may cause more HIV new infections and more undiagnosed infections due to more and more HIV-positive individuals and/or AIDS patients being accumulated. As the green curve shows, the number of new infections has the greatest value when peaking compared with other two curves. Figure 5b and Table 2 present the comparisons between the undiagnosed infections for three different groups of progression rates. For different progression rates, both the peak values of new and undiagnosed infections may appear at different years. For instance, new infections peak at year 2008 and undiagnosed infections reach the peak at year 2006 for PR2. So, both new infections and undiagnosed infections could peak between 2006 and 2008. At the mean time, great diagnosis probabilities in all three CD4 stages are associated with large progression rates, i.e. short waiting time, as shown in Fig. 5c, in which the diagnosis probabilities in three CD4 stages for three different groups of progression rate are illustrated. It follows from Fig. 5a, b and c that the greater progression rates, i.e. shorter waiting time, corresponds to the less new infections and undiagnosed infections and larger diagnosis probabilities, while the epidemic situation will become severer for the slower disease progression (i.e., the smaller progression rates).

We repeat estimating new infections, undiagnosed infections by using method 2. If PR1 is initially chosen, the estimated new HIV infections, undiagnosed infections and diagnosis rates from 2005 to 2017 are illustrated in Fig. 4b, d and f, respectively. All of the three estimated curves show similar shapes with those obtained by method 1, as shown in Fig. 4a, c and e. The number of new infections peaks at year 2006 to 2008, and decreases from then on. The undiagnosed infections increases gradually from year 2005 to 2009, then decreases until 2017. Meanwhile, the diagnosis probability at late CD4 stage, i.e., less than 350, decreases slowly from 2006, while the diagnosis probabilities at early CD4 stage, i.e. [500,*∞*), grows gradually from 2006 to 2017. Also, there are jumps for diagnosis probabilities at all the three CD4 stages in 2005 to 2011. Comparing with those plotted by method 1, both new HIV infection and diagnosis rate curves on method 2 are smoother with less oscillations. Nevertheless, the number of the estimated undiagnosed infections are slightly smaller, while the diagnosis rates are a bit greater than those obtained by method 1, as shown in Tables 1, 2 and 3, in which estimations by two methods are listed in parallel. Also, if we change the progression rate and conduct the estimation procedure again, the new infections, undiagnosed infections and diagnosis rates vary similarly as we observed by method 1, see Tables 1, 2 and Additional file 5 for details.

## Discussions

In this study, based on CD4-staged structure, we formulated two CD4-staged models (Markov model named as method 1 and deterministic compartmental model named method 2) to obtain the declining trend of new and undiagnosed HIV infections but late diagnosis of the undiagnosed HIV cases, which constitute the feature of HIV infections in Guangxi. Given the fact that HIV infected cases with CD4 level lower than 200 are also defined as AIDS patients in China, we extend the CD4-staged structure model used by Birrell et al. [30] and Van Sighem et al. [31] by merging these two groups together and only considering three CD4 stages, i.e. [500,*∞*),[350,500) and [200,350). This modelling approach can link the CD4 data at the individual level to the surveillance data at the population level to some what and make a relatively accurate estimation on new infections and undiagnosed infections.

The annually reported data from the HIV/AIDS information system reveal that the number of newly reported HIV and AIDS cases began to decrease from 2011 after 15 years of increasing. More and more infected individuals receive CD4 test shortly after diagnosed with HIV, and the proportion of individuals linked with CD4 data reached 85% in 2017. However, still half of the cases linked with CD4 data were diagnosed at late CD4 stage, consistent with the former results [17], which causes a high potential risk of HIV transmissions.

The predicated new infections, based on our estimations by method 1, began to decrease from the period between 2006 and 2008, but rebound for a short term around year 2011-2012. Similarly, the undiagnosed infections increases straightly until the period of 2006 and 2008, and then decreases gradually until 2017 but with a slightly less declining slop in 2012. The diagnosis probabilities keep at low values at three CD4 stages and show no much changes over time with slight decrease at the late CD4 stage and some increase at the early CD4 stage, and moreover the undiagnosed infected cases have large probabilities to be diagnosed at late CD4 stage. Compared with the first method, similar estimation results were obtained by the second method, but the estimation curves were much smoother, while the number of undiagnosed infections are smaller and the diagnosis rates are larger. Less oscillations of the estimation curves indicate that method 2 may be more stable and less affected by the stochastic factors, nevertheless, method 2 may in the meantime ignore the normal fluctuations of the data, while method 1 may in the opposite lead to the over-fitting phenomenon. Note that we only know the diagnosis time, rather than the infection time of the HIV infected cases, then we do not have the real data about the number of new infections and the CD4 level of new infections. Thus, it is impossible to compare the estimated number of new infections and the real new infections, so is the CD4 level of new infections. But the data fitting results tell us that the model predicted results for both annually new HIV/AIDS diagnosis and the number of cases in each CD4 stage are very close to the real data, as shown in Additional files 2 and 3, which indicates that the estimation results can reflect the real situation well.

By combining the two groups of the estimation results we conclude that the decreasing trend in new and undiagnosed infections, and late diagnosis of the undiagnosed cases are two major features for HIV epidemics in Guangxi, China. The declining trend in estimated HIV new infections is certainly encouraging, and may be attributed to all kinds of interventions strengthened in recent years [4–6, 13], such as the NEP, MMT, HIV testing expanding, antiretroviral treatment, condom promotion, and AIDS awareness work. While low diagnosis rate and late diagnosis may be caused by complexity of transmission dynamics including the diversified and interlaced transmission routes, complicated risk groups and etc in Guangxi. Since sexual transmission has been the main route of HIV transmission from 2006 [11], HIV infected cases with age of 50 years old or older account for a large proportion in Guangxi [10], hence old population may greatly affect the HIV epidemic in Guangxi. There is an evidence [11, 36] showing that HIV prevalence increases among old age clients, whose protection rate when having commercial sex is only less than 14%, which is relatively low compared with the young clients in China [37–39], and the knowledge about HIV/AIDS is also much lower compared with other population [36, 40]. Furthermore, the proportion who have ever got HIV test is extremely low compared with the young population [36, 41]. These directly result in the late diagnosis of HIV among old population [42]. Further, a study conducted from 1998 to 2010 in Liuzhou, a city in Guangxi, found that the genetic subtypes of HIV were CRF01-AE for most of the new diagnosed HIV infections [43]. Another study revealed that subtypes CRF01-AE may cause the CD4 counts decreasing with a faster speed within hosts [44], this may be another reason for the late diagnosis of HIV infections in Guangxi. Therefore, the decision-makers are suggested to allocate more resources to identify HIV infections at their early CD4 stage among high risk populations like clients over 50 years old or the MSMs. Meanwhile, comprehensive intervention measures should be continually strengthened in avoid of the rebound of new infections.

Since we have no liable data or enough information on the progression rates among different stages in Guangxi, three groups of progression rates from references [30–32] were adopted to investigate the variation in new and undiagnosed infections with progression rates. It is worth mentioning that progression rates do affect the estimation of HIV new infection, and generally speaking, the longer period the HIV infected individuals stay in a state the more new infections estimated, or alteratively quick disease progression may induce relatively low new infections. As the study showed that CRF01-AE is the main genetic subtype of HIV in Guangxi [43], and this subtype always leads to fast HIV progression [44]. Thus, it is possible that HIV cases in Guangxi has a much quicker disease progression, as a result the new infections may decline at a much quicker way in Guangxi. However, this could only be confirmed when the reliable progression rates are available. We leave this for the future work. Note that this study is a bit geographically specific, since the data for Guangxi used here is more complete and regular than those for the mainland China, but we hope the approaches we used are able to be applied more generally.

There are limitations for the methods used in this article. Firstly, the two methods are in fact back-calculation method, thus, could only“back-calculate" the new infections and undiagnosed cases as well as the diagnosis rates in past years. Hence, it is difficult to make predictions. Extending work is needed so as to predict the future epidemic trend. Secondly, as has been mentioned in the main text, estimations of new infections in the last few years have large uncertainties. More accurate estimates could be obtained once we have more available data by methods used in this study. In recent years, biomarker approach has been proposed by researchers [45–47], which may help to identify recent infections and then give estimations of new infection. We leave this for future work.

## Conclusions

The estimated new and undiagnosed infections in Guangxi began to decrease in recent years, which means that HIV/AIDS epidemic in Guangxi has been controlled to a certain extent. While the diagnosis rate still needs to be improved as the estimated diagnosis probabilities/rates keep at a very low level and a large number of infections are undiagnosed. More attentions should be paid to identify infections at their early CD4 stages. Meanwhile, comprehensive intervention measures should be continually strengthened in avoid of the rebound of new infections.

## Supplementary information

**Additional file 1** Descriptions of Method 1 and Method 2.

**Additional file 2** Comparisons between real data and model predictions obtained by method 1.

**Additional file 3** Comparisons between real data and model predictions obtained by method 2.

**Additional file 4** Yearly new infections, undiagnosed prevalence, and diagnosis probabilities from 1990 to 2017 (estimated by method 1).

**Additional file 5** The comparison of new infections, undiagnosed infections, and diagnosis rates by three different groups of progression rates (estimated by method 2).

## Data Availability

The data that support the findings of this study are available from Guangxi Center for Disease Control and Prevention but restrictions apply to the availability of these data, which were used under license for the current study, and so are not publicly available. Data are however available from the authors upon reasonable request and with permission of Guangxi Center for Disease Control and Prevention.

## Abbreviations

HIV: Human Immunodeficiency Virus
AIDS: Acquired Immune Deficiency Syndrome
NEP: Needle Exchange Programme
MMT: Methadone Maintenance Treatment
GACP: Guangxi AIDS Conquering Project
MSM: Men Who Have Sex with Men
PR: Progression Rate
