# Supplementary material for: Declining trend in HIV new infections in Guangxi, China: insights from linking reported HIV/AIDS cases with CD4-at-diagnosis data
Source: BMC Public Health. 2020 Jun 12;20:919. doi: 10.1186/s12889-020-09021-9 (PMC7290136; doi:10.1186/s12889-020-09021-9)
Supplement: Supplementary file 1 — Additional file 1 Descriptions of Method 1 and Method 2. [file 12889_2020_9021_MOESM1_ESM.pdf]

Methods

We apply the methods proposed by Birrell et al. [1] and Sighem et al. [2], respectively. As the model structures in this article contain only three CD4 stages, which are different with those used by Birrell et al. and Sighem et al., and our estimation procedure in the second method is slightly different with that proposed by Sighem et al. [2], we summarize the main ideas of the methods as follows.

Method 1

Using the similar notations to those in Birrell's method [1], we let h_j be the expected number of new infections in the time interval $[t_{j-1}, t_j]$. Let $\rho_{k,k+1}$ be the progression probability between disease stages which are supposed to be known, and let $\boldsymbol{d}_j = (d_{1,j}, \cdots, d_{3,j})$, where $d_{k,j} = d_k(t_j)$ denotes the diagnosis probability at CD4 stage k in the time interval $[t_{j-1}, t_j]$, $j = 1, \cdots, N$, $k = 1, 2, 3$. Then, the expected number of undiagnosed infected individuals at CD4 stage k in the time interval $[t_{j-1}, t_j]$ can be obtained and denoted by $\boldsymbol{e}_j = (e_{1,j} \cdots, e_{3,j})$. Furthermore, the expected number of new HIV and AIDS diagnosis μ_j^{HIV} and μ_j^{AIDS} in the time interval $[t_{j-1}, t_j]$ can be calculated according to the model.

$$\begin{aligned} \mu_j^{HIV} &= \mathbf{e_{j-1}} \cdot \mathbf{d_j}^T, \text{and} \\ \mu_j^{AIDS} &= e_{3,j}(1 - d_{3,j})\rho_{3,4} \end{aligned} \tag{S.1}$$

where

$$\mathbf{e_j} = \mathbf{P_j^T} \mathbf{e_{j-1}} + (h_j, 0, 0, 0)^T.$$

and $\mathbf{P_j}$ is the transition matrix which describes the proportion of individuals transit between different stages. Then,

$$(\mathbf{P_j})_{k,l} = \begin{cases} (1 - d_{k,j})(1 - \rho_{k,k+1}) & k = l, \\ (1 - d_{k,j})\rho_{k,k+1} & k = l - 1, \\ 0 & \text{otherwise.} \end{cases} \tag{S.2}$$

It is supposed that the numbers of new diagnosed HIV and AIDS individuals follow the Poisson distributions with means $\{\mu_j^{HIV} : j = 1, \cdots, N\}$ and $\{\mu_j^{AIDS} : j = 1, \cdots, N\}$,

respectively. Then, the likelihood function yields

$$L_1(\mathbf{D}, \mathbf{A}; \mathbf{h}, \mathbf{d}) \propto \prod_{j=1}^{N} (\mu_j^{AIDS})^{A_j} \exp(-\mu_j^{AIDS}) \times (\mu_j^{HIV})^{D_j} \exp(-\mu_j^{HIV}),$$

where $\boldsymbol{D} = \{D_j : j = 1, \cdots, N\}$, $\boldsymbol{A} = \{A_j : j = 1, \cdots, N\}$ are the observed new HIV and AIDS diagnosis. Let $\boldsymbol{C}_j = (C_{1,j}, C_{2,j}, C_{3,j})$, where $C_{k,j} = C_k(t_j)$, be the number of newly observed infected cases with CD4-at-diagnosis falling into each CD4 group, and $\boldsymbol{N}_j = \sum_k^3 C_{k,j}$ is the number of observed cases linked with CD4 counts at diagnosis. It is assumed that $\boldsymbol{C}_j$ follows the multinomial distribution, then

$$\boldsymbol{C}_j \sim \text{Multinomial}(\boldsymbol{N}_j, \boldsymbol{r}_j),$$

$$\boldsymbol{r}_j = \{r_{k,j} : k = 1, 2, 3\}, \qquad r_{k,j} = \frac{e_{k,j-1} d_{k,j}}{\mu_j^{HIV}}, j = 1, \cdots, N,$$

where the likelihood contributed by the CD4-at-diagnosis data gives

$$L_2(\boldsymbol{C} | \boldsymbol{D}; \boldsymbol{h}, \boldsymbol{d}) \propto \prod_{j=1}^{N} \prod_{k=1}^{3} r_{k,j}^{C_{k,j}}.$$

The full likelihood follows

$$L(\boldsymbol{D}, \boldsymbol{A}, \boldsymbol{C}; \boldsymbol{h}, \boldsymbol{d}) = L_1(\boldsymbol{D}, \boldsymbol{A}; \boldsymbol{h}, \boldsymbol{d}) L_2(\boldsymbol{C} | \boldsymbol{D}; \boldsymbol{h}, \boldsymbol{d}).$$

By using the hierarchical Bayesian approach which includes a random walk specification for the incidence and diagnosis curves, we can estimate the number of new infections and diagnosis probabilities at each CD4 stage in each time interval, and the undiagnosed prevalence. That is both the number of new infections and diagnosis probabilities have autocorrelation, i.e. $\gamma_i \sim N(\gamma_{i-1}, \sigma_\gamma^2)$ and $\delta_{k,j} \sim N(\delta_{k,j-1}, \sigma_{\delta,k}^2)$, where $\gamma_i = log(h_i)$ and $\delta_{k,j} = logit(d_{k,j})$. A factor c_{90} is introduced to represent the reduced diagnosis before 1995, because national sentinel surveillance began to be implemented from 1995 [3].

$$d_{k,i} \quad = \quad c_{90} d_{k,6}, \quad i = 1, \cdots, 6 \text{ (pre-1995 diagnoses)}$$

Method 2

A deterministic model is proposed based on the CD4-staged structure shown in Figure 1, In this model, let $\mathcal{U}_k(t)$, $k = 1, 2, 3$, denote the number of undiagnosed HIV-positives with

CD4 counts corresponding to $[500, \infty)$, $[350, 500)$ and $[200, 350)$ at time t, $\mathcal{A}(t)$ be the accumulative AIDS diagnosis (not include those who were diagnosed in HIV stage and then progressed to AIDS stage), $\mathcal{D}_k(t)$ and $\mathcal{C}_k(t)$, $k = 1, 2, 3$, be the corresponding accumulative diagnosed HIV-positives and HIV-positives who have CD4 count measurements within 3 months after diagnosis in each CD4 stage, respectively. To be consistent with method 1, we let $\rho_{k,k+1}$, $k = 1, 2, 3$ and $d_k(t)$ be the progression rate from stage k to $k+1$ and diagnosis rate at stage k, respectively. $q(t)$ is the probability for HIV infected cases having CD4 measurements within 3 months after diagnoses at time t. Then the model equations can be described as follows.

$$
\begin{aligned}
\frac{\mathrm{d}\mathcal{U}_1(t)}{\mathrm{d}t} &= h(t) - \rho_{12}\mathcal{U}_1(t) - d_1(t)\mathcal{U}_1(t) - \mu\mathcal{U}_1(t),\\
\frac{\mathrm{d}\mathcal{U}_2(t)}{\mathrm{d}t} &= \rho_{12}\mathcal{U}_1(t) - \rho_{23}\mathcal{U}_2(t) - d_2(t)\mathcal{U}_2(t) - \mu\mathcal{U}_2(t),\\
\frac{\mathrm{d}\mathcal{U}_3(t)}{\mathrm{d}t} &= \rho_{23}\mathcal{U}_2(t) - \rho_{34}\mathcal{U}_3(t) - d_3(t)\mathcal{U}_3(t) - \mu\mathcal{U}_3(t),\\
\frac{\mathrm{d}\mathcal{A}(t)}{\mathrm{d}t} &= \rho_{34}\mathcal{U}_3(t),\\
\frac{\mathrm{d}\mathcal{D}_1(t)}{\mathrm{d}t} &= d_1(t)\mathcal{U}_1(t),\\
\frac{\mathrm{d}\mathcal{D}_2(t)}{\mathrm{d}t} &= d_2(t)\mathcal{U}_2(t),\\
\frac{\mathrm{d}\mathcal{D}_3(t)}{\mathrm{d}t} &= d_3(t)\mathcal{U}_3(t),\\
\frac{\mathrm{d}\mathcal{C}_1(t)}{\mathrm{d}t} &= q(t)d_1(t)\mathcal{U}_1(t),\\
\frac{\mathrm{d}\mathcal{C}_2(t)}{\mathrm{d}t} &= q(t)d_2(t)\mathcal{U}_2(t),\\
\frac{\mathrm{d}\mathcal{C}_3(t)}{\mathrm{d}t} &= q(t)d_3(t)\mathcal{U}_3(t).
\end{aligned}
\tag{S.3}
$$

Since new infections and diagnosis rate may not always change much within successive years, both new infections $h(t)$ and diagnosis rate $d_i(t)$, $i = 1, 2, 3$ are supposed to be step functions, which can also reduce the number of parameters to be estimated. The step

functions of new infections and diagnosis rates are given as follows:

$$h(t) = \begin{cases} \hat{h}_1, & \text{for } 1990 \leq t < 1994, \\ \hat{h}_2, & \text{for } 1994 \leq t < 1997, \\ \hat{h}_3, & \text{for } 1997 \leq t < 2000, \\ \cdots & \cdots \\ \hat{h}_8, & \text{for } 2012 \leq t < 2015. \\ \hat{h}_9, & \text{for } 2015 \leq t. \end{cases} \quad d_i(t) = \begin{cases} \hat{d}_{i1}, & \text{for } 1990 \leq t < 1996, \\ \hat{d}_{i2}, & \text{for } 1996 \leq t < 2000, \\ \hat{d}_{i3}, & \text{for } 2000 \leq t < 2005, \\ \hat{d}_{i4}, & \text{for } t = 2005, \\ \hat{d}_{i5}, & \text{for } 2006 \leq t < 2011, \\ \hat{d}_{i6}, & \text{for } t = 2011, \\ \hat{d}_{i7}, & \text{for } 2012 \leq t < 2016, \\ \hat{d}_{i8}, & \text{for } 2016 \leq t. \end{cases} \tag{S.4}$$

with positive constants $\hat{h}_i (i = 1, \cdots, 9)$ and $\hat{d}_{ij} (j = 1, \cdots, 8)$.

References

[1] Birrell PJ, Chadborn TR, Gill ON, Delpech VC, De Angelis D. Estimating trends in incidence, time-to-diagnosis and undiagnosed prevalence using a CD4-based Bayesian back-calculation. Statistical Communications in Infectious Diseases. 2012; 4(1).

[2] van Sighem A, Nakagawa F, De Angelis D, Quinten C, Bezemer D, de Coul EO, et al. Estimating HIV incidence, time to diagnosis, and the undiagnosed HIV epidemic using routine surveillance data. Epidemiology. 2015; 26: 653-660.

[3] Wu Z, Sullivan SG, Wang Y, Rotheram-Borus MJ, Detels R. Evolution of Chinas response to HIV/AIDS. Lancet. 2007; 369(9562): 679-690.
