# Supplementary material for: Declining trend in HIV new infections in Guangxi, China: insights from linking reported HIV/AIDS cases with CD4-at-diagnosis data
Source: BMC Public Health. 2020 Jun 12;20:919. doi: 10.1186/s12889-020-09021-9 (PMC7290136; doi:10.1186/s12889-020-09021-9)
Supplement: Supplementary file 2 — Additional file 2 Comparisons between real data and model predictions obtained by method 1. [file 12889_2020_9021_MOESM2_ESM.pdf]

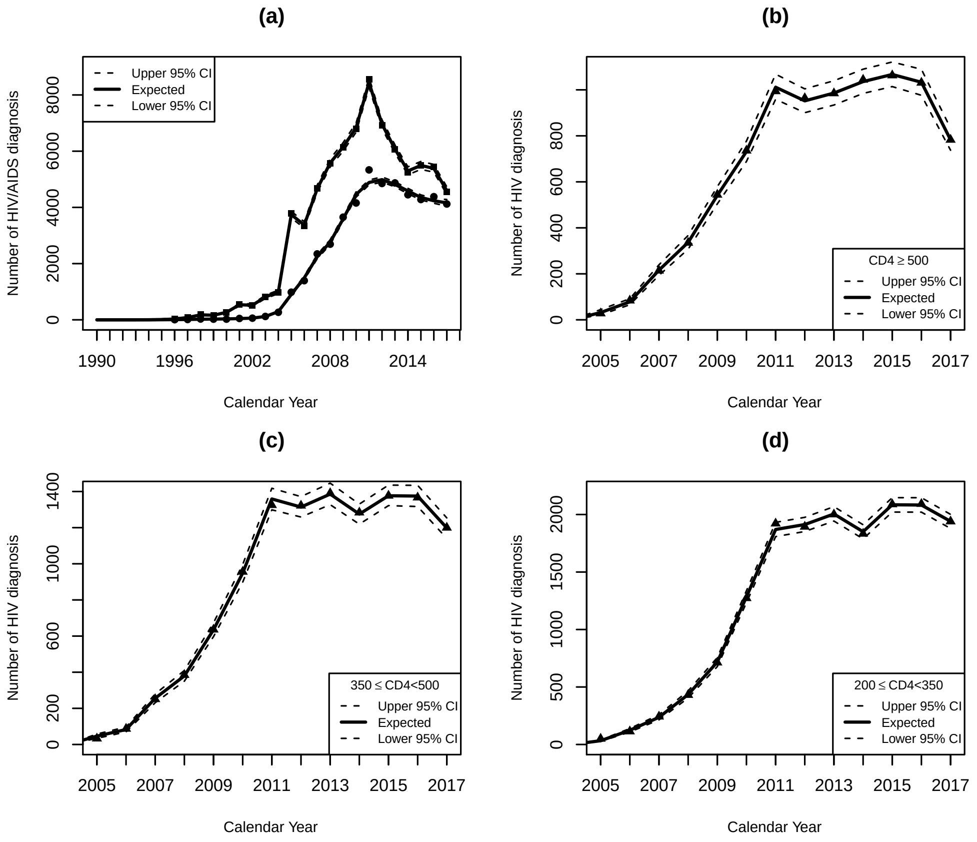

Fig. S1: Comparisons between real data and model predictions obtained by method 1. (a) newly HIV and AIDS diagnosis; (b) newly observed cases at CD4 stage $[500, \infty)$; (c) newly observed cases at CD4 stage $[350, 500)$; (d) newly observed cases at CD4 stage $[200, 350)$. Dark squares, circles and triangles denote the observed values. The dotted lines give the 95% CI. Progression rate 1 is adopted. The dark squares, circles and triangles describe the newly diagnosed HIV cases, AIDS patients and HIV cases at each CD4 stage, while the dotted lines show the 95% credible intervals of the posterior predictive distribution of the data. Most of the data are fitted well and covered by the 95% credible interval. It is noticeable the 95% credible intervals for HIV and AIDS diagnosis data are very narrow, while those for HIV cases at each CD4 stage are rational. This is mostly because the values of HIV and AIDS diagnosis data are too large.
