# Supplementary material for: Declining trend in HIV new infections in Guangxi, China: insights from linking reported HIV/AIDS cases with CD4-at-diagnosis data
Source: BMC Public Health. 2020 Jun 12;20:919. doi: 10.1186/s12889-020-09021-9 (PMC7290136; doi:10.1186/s12889-020-09021-9)
Supplement: Supplementary file 4 — Additional file 4 Yearly new infections, undiagnosed prevalence, and diagnosis probabilities from 1990 to 2017 (estimated by method 1). [file 12889_2020_9021_MOESM4_ESM.pdf]

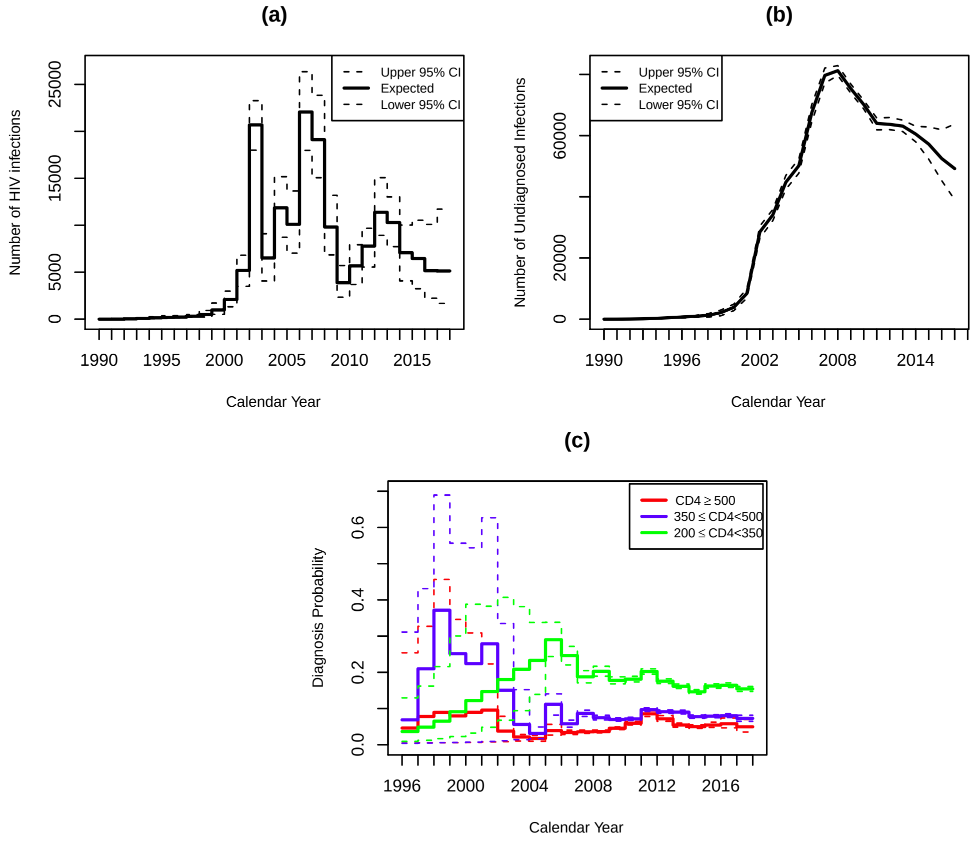

Fig. S3: Yearly new infections (a) undiagnosed prevalence (b) and diagnosis probabilities (c) from 1990 to 2017 (estimated by method 1). The dotted lines give the 95% CI.
