# Supplementary material for: Declining trend in HIV new infections in Guangxi, China: insights from linking reported HIV/AIDS cases with CD4-at-diagnosis data
Source: BMC Public Health. 2020 Jun 12;20:919. doi: 10.1186/s12889-020-09021-9 (PMC7290136; doi:10.1186/s12889-020-09021-9)
Supplement: Supplementary file 5 — Additional file 5 The comparison of new infections, undiagnosed infections, and diagnosis rates by three different groups of progression rates (estimated by method 2). [file 12889_2020_9021_MOESM5_ESM.pdf]

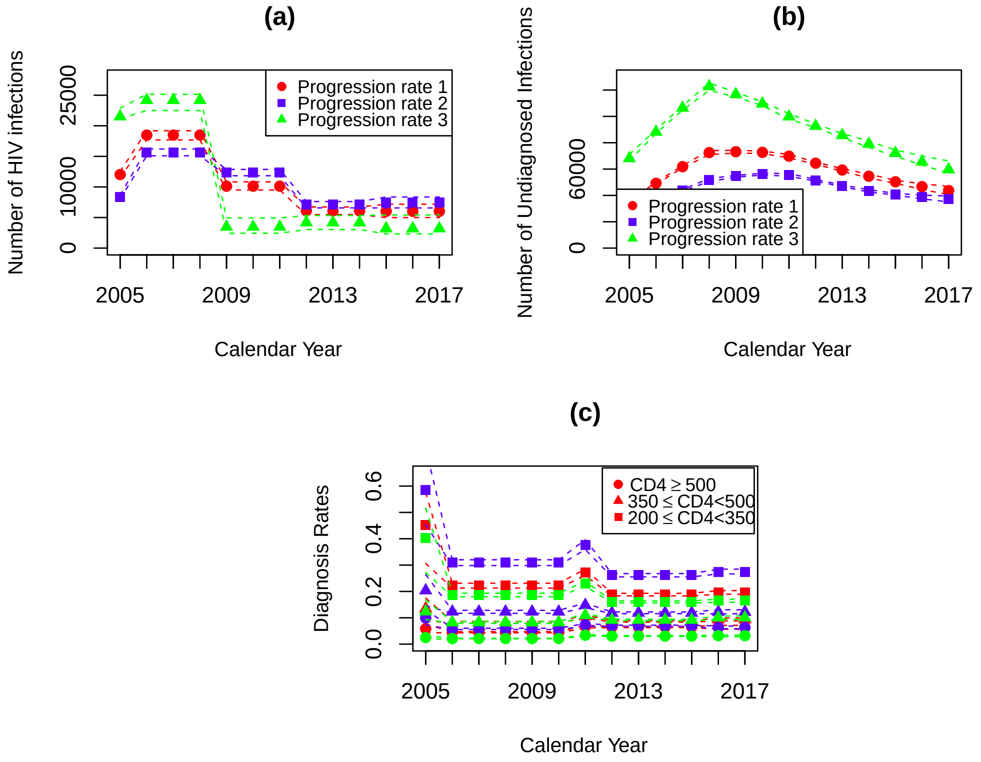

Fig. S4: The comparison of new infections (a), undiagnosed infections (b), and diagnosis rates (c) by three different groups of progression rates (estimated by method 2). The waiting time in each CD4 stage for red, blue and green are PR1:3, 3, 3; PR2: 2.56, 2.17, 2.16 and PR3: 6.37, 2.86 3.54, respectively. The dotted lines give the 95% CI.
